# Neurodevelopmental challenges at age four following fetal exposure to maternal opioid maintenance treatment

**DOI:** 10.1038/s41390-025-04029-6

**Published:** 2025-03-27

**Authors:** Minna M. Kanervo, Sarimari J. Tupola, Eeva M. Nikkola, Mika Gissler, Hanna K. Kahila, Krista M. Rantakari

**Affiliations:** 1 University of Helsinki Doctoral School, Helsinki, Finland; 2 Children’s Hospital, Helsinki University Hospital, Pediatric Research Center, and University of Helsinki, Helsinki, Finland; 3 THL, Department of Data and Analytics, Finnish Institute for Health and Welfare, Helsinki, Finland; 4 Karolinska Institutet, Department of Molecular Medicine and Surgery, Stockholm, Sweden and Region Stockholm, Academic Primary Health Care Centre, Stockholm, Sweden; 5 Gynecology and Obstetrics, Helsinki University Hospital and University of Helsinki, Helsinki, Finland

## Abstract

**Background:**

The impact of intrauterine exposure to maternal opioid use disorder (OUD) and opioid maintenance treatment (OMT) on child development is not fully understood. This population-based cohort study investigated the neurodevelopmental and behavioral outcomes of four-year-old children prenatally exposed to maternal OMT, hypothesizing greater challenges compared to their same-aged peers in Finland.

**Methods:**

Children with intrauterine exposure to buprenorphine±naloxone or methadone (*n* = 123) were compared with typically developing children (*n* = 434) using standardized language, motor-perceptual, and attention-behavioral skills screening tests. ICD-10 diagnoses for developmental and behavioral disorders were compared with national data from 50,457 Finnish children. Odds ratios (OR) and 95% confidence intervals (CI) were calculated to assess risks.

**Results:**

Children with prenatal OMT exposure exhibited significantly higher rates of developmental challenges as indicated by screening tests and ICD-10 diagnoses, including speech and language disorders, ADHD, conduct, emotional, and social disorders (F80, F90-94), with *ORs* ranging from *8.97 to 210.21*. Additional risk factors included male sex (*p* < *0.001*), methadone (*p* = *0.004*), illicit drug exposure (*p* = *0.011*), and domestic violence (*p* = *0.032*).

**Conclusion:**

Children born to mothers with OUD and OMT face significantly elevated risks of developmental and behavioral challenges. Close monitoring, stable environment and early support for these children with multiple risk factors are crucial.

**Impact:**

- This population-based cohort study demonstrates that children with in-utero exposure to maternal OMT are at high risk of neurodevelopmental, emotional and behavioral difficulties at age four.
- Our results particularly add new knowledge of specific domains (skills) in neurodevelopmental screening tests and ICD-10 diagnoses in these children.
- Beyond OMT, multiple additional risk factors such as fetal exposures to illicit substances or other harmful substances and postnatal environmental instability further compound the likelihood of later-life impairments.
- These findings emphasize the critical need for comprehensive follow-up, stable environment, and early interventions for this vulnerable group.

## Introduction

While the effects of intrauterine opioid exposure on children remain incompletely understood, opioid maintenance treatment (OMT) with buprenorphine or methadone is recommended during pregnancy to mitigate illicit drug use and support better life management.^1^ Opioids readily cross the placenta potentially affecting the developing brain and overall health of the fetus. Some evidence suggests that children exposed to opioids prenatally may have increased risks for language^2–4^ and motor development delays.^5,6^ Additionally, children who experience neonatal opioid withdrawal syndrome (NOWS) may experience even more severe challenges.^7–10^ Research also indicates that boys may be more susceptible to developmental difficulties than girls.^4,11^ Environmental factors, including non-sensitive or non-responsive parenting, may also impair language skills or neurodevelopment.^12–14^ However, some studies suggest that intrauterine opioid exposure may not necessarily lead to impaired neurodevelopment.^15–18^ Children’s developmental trajectories are also significantly influenced by their environment and exposure to adverse childhood experiences (ACE).^19^

This study investigated the impact of intrauterine exposure to maternal OMT on children’s neurological development and behavior, focusing primarily on developmental delays and diagnoses at the age of four. Based on previous studies and clinical experience, we hypothesized that children exposed to intrauterine OMT would experience more developmental, emotional and behavioral challenges than same-aged Finnish children from the general population.

## Methods

### Patients

**Fig. 1:**
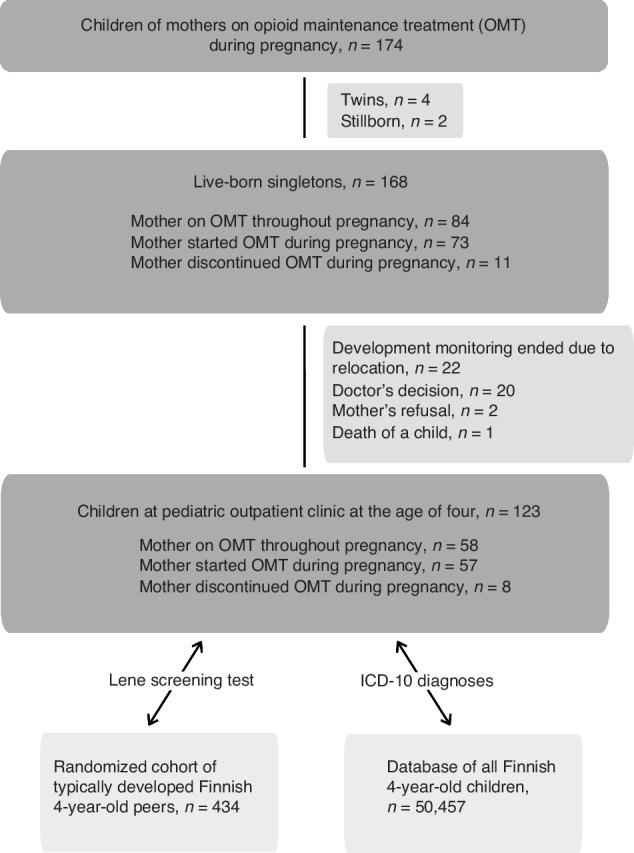
Study flowchart. Out of 174 children with intrauterine exposure to maternal opioid maintenance treatment, after exclusion (*n* = 6) and dropouts (*n* = 22), 123 continued to visit the pediatric outpatient clinic at the age of 4 years. Their test performances and diagnostic frequencies were compared to those of a cohort of 434 typically developing children and 50,457 Finnish children of the same age.

This population-based cohort study examined children born to mothers who received OMT during pregnancy in the Helsinki metropolitan area, Finland, with a population of 1.7 million. The study included children of 172 mothers born between January 1, 2011, and December 31, 2018 (Fig. 1), representing 0.15% of 119,200 births during this period. After excluding multiple and stillbirths,168 livebirth singletons were eligible for analysis. These children were initially treated in the neonatal unit and then monitored at the outpatient clinic for four years at the Children’s Hospital of Helsinki University Hospital. Reference data for Finnish parturients and newborns were obtained from perinatal statistics based on the Medical Birth Register.

During follow-up, 22 children moved to another municipality, 20 completed follow-up per clinical decision, and two mothers discontinued participation. One child died at seven weeks due to sudden infant death syndrome. Consequently, 123 children (73% of the initial cohort) remained in the final dataset at age four. Participants whose mothers received the same non-changed OMT throughout their pregnancies were included in our previous publications.^20–22^

This research followed the ethical requirements of the Helsinki University Hospital. The protocols were approved by the Hospital District of Helsinki and Uusimaa (HUS/54/2019/Feb/4/2019).

### Outcomes

The primary outcomes were developmental difficulties identified in standard screening tests described below and diagnoses according to the 10th Revision of the International Statistical Classification of Diseases and Related Health Problems, (ICD-10) in four-year-old children with in-utero exposure to maternal OMT, compared to same-aged children of the Finnish general population.

Developmental difficulties were assessed with the *Lene* screening test. *Lene* is an abbreviation for *Le*ikki-ikäisen lapsen *ne*urologinen testi (Finnish, Neurological test of a toddler). It is a routine neurodevelopmental screening test for preschool children administered at ages 2.5, 3, 4, 5, and 6 years It was created by a multidisciplinary child neurology team in the 1990s and remains in use unchanged. The test includes age-appropriate assessments derived from various standardized tests and is conducted in Finland during well-child visits.^23^ The items included in the four-year-old children’s *Lene* test assess interactional, attentional, play, and self-help skills; expressive speech; understanding instructions and questions; counting; color naming; auditory discrimination; walking on toes, standing on one foot, hopping, and ball skills; copying designs; block construction; stringing beads, and cutting with scissors. These items are grouped into domains of language, motor-perceptual, and attention-behavioral skills, with results categorized as passed (normal) or failed (difficulties noted). Refusal to complete the test was categorized as failed due to its association with poorer performance.^23^

The screening results of the cohort were compared with a reference group of 434 randomly selected Finnish four-year-olds from routine health clinic data.^23^ The reference group, data comprising 197 males and 237 females, underwent Lene screening tests in 2001. The parents’ educational level reflected the average for the population; fewer than ten percent had not completed nine years of basic education, 62% had advanced vocational education, and 12% held a university degree. Data on the children’s intrauterine exposure to alcohol, smoking, or drugs were not collected.^23^

Data on chronic diagnoses of OMT-exposed children at the age of four were collected from the hospital medical records. Specialists in child neurology, phoniatrics, and child psychiatry assessed the diagnoses according to appropriate diagnostic processes and criteria. ICD-10 diagnosis codes from specialized health care, as registered in the Hospital Discharge Register, were used to compare the prevalence of developmental (F80–F89, Q86.0) and behavioral/emotional (F90–F98) disorders with those in a national cohort of 50,457 Finnish four-year-olds born in 2018.

### Covariates

Child covariates included birth data: sex as defined at birth by external characteristics, gestational age, birth measurements (weight, length and head circumference), umbilical artery pH and base excess, Apgar scores, any medical diagnoses, and need for pharmacological treatment for NOWS, medication, and duration of the medication. NOWS symptoms were assessed with Finnegan scores^24^ along with drug screenings. Data on subsequent development, somatic health, growth, and living arrangements (whether with biological parents at home or in a parental rehabilitation unit, or out-of-home care) were collected at two months, one year and annually up to the age of four.

Maternal covariates included (1) maternal background (age, height, ethnicity, parity); (2) OMT medication and dosage; (3) other diagnoses and medications; (4) illicit drug use, smoking, and alcohol intake; (5) pregnancy complications and delivery details; (6) OMT adherence after the childbirth, and (7) death. Data on smoking, alcohol, and substance use were based on self-reports and voluntary urine drug tests. Father-related data included living arrangements, substance use disorder, OMT, and death, reported by either father or mother.

### Statistical analysis

We analyzed anonymized data using SPSS version 29 (IBM Corp, New York, New York). Pearson’s chi-square tests were used to calculate Odds ratios (OR) and 95% confidence intervals (CI) for Lene results and ICD-10 diagnoses, comparing the study cohort with Finnish four-year-olds. All variables were tested as a group within the study cohort and compared to reference groups. Additionally, we conducted subgroup analyses within the OMT groups. To identify statistical associations, we used Pearson’s chi-square tests and Fisher’s exact tests for categorical and dichotomous variables; and univariate analysis, Mann-Whitney U tests, or Kruskal-Wallis tests for continuous variables. Covariates were checked for spurious associations as needed. Bonferroni adjustments were applied for post-hoc analyses. All statistical tests were two-tailed, with statistical significance set at *p*-value < 0.05.

## Results

### At birth

As shown in Table 1, 95% of the children were born full-term. The newborns were generally in good condition, with no significant differences compared to the general Finnish population, except for relatively small birth size.

**Table 1:** Characteristics of children at birth and related to fetal phase.

*n* (%) or mean (range)	Cohort, *n* = 123	Finland, (*n* = 45,056)^a^	OR (95% CI)	*p*
Birth				
Mother’s first child (primipara)	53 (43)	19,689 (44)	0.98 (0.68 to 1.39)	0.8
Mode of the delivery				
Vaginal	88 (72)	31,810 (71)	1.05 (0.71 to 1.55)	0.8
Instrumental	4 (3)	4415 (10)	0.31 (0.11 to 0.84)	0.01
Cesarean section	31 (25)	8831 (20)	1.38 (0.92 to 2.08)	0.1
Sex: male	65 (53)	23,063 (51)	1.07 (0.75 to 1.52)	0.7
Gestational age (weeks)	39 + 5 (26 + 0 to 42 + 3)	39 + 6 (22 + 1 to 44 + 1)		0.5
Premature ( < h 37 + 0)	6 (5)	2531 (6)	0.86 (0.38 to 1.96)	0.7
Apgar 1 min	9 (3 to 10)	9 (0 to 10)		0.4
Apgar 5 min	9 (5 to 10)	9 (0 to 10)		0.2
Umbilical artery pH	7.25 (7.05 to 7.41)	7.24 (6.81 to 7.74)		0.2
Birthweight (g)	3257 (925 to 4790)	3498 (410 to 5520)		<0.001
Birthweight (SD)	−0.8 (−3.8 to 2.3)			
Neonatal length (cm)	49 (33 to 55)	50 (25 to 62)		0.001
Neonatal length (SD)	−0.9 (−5.6 to 2.2)			
Neonatal HC (cm)	34 (23 to 39)	35 (19 to 49)		<0.001
Neonatal HC (SD)	−0.6 (−3.6 to 3.6)			
SGA	27 (22)	4505 (10)	2.53 (1.65 to 3.88)	<0.001
Pharmacotherapy for NOWS	69 (56)			
Morphine	67 (97)			
Morphine and phenobarbital	2 (3)			
Duration of treatment for NOWS (days)	18 (5 to 102)			
Congenital diagnoses during the neonatal period	7 (6)	2277 (5)^b^	1.25 (0.58 to 2.69)	0.5
Skeletal	4 (3)^c^			
Urinary tract	2 (1.6)			
Gastrointestinal	1 (0.8)^c^			
Mother / fetal phase				
Duration of maternal OMT before pregnancy				
<1 year	13 (22)			
1-4 years	31 (51)			
5 years or longer	16 (27)			
First visit to maternal outpatient clinic				
I trimester	67 (55)			
II trimester	43 (35)			
III trimester	12 (10)			
Fetal exposure to maternal OMT				
Bup/Nx throughout	46 (37)			
Methadone throughout	9 (7)			
Methadone switched to Bup/Nx	3 (2)			
Bup/Nx started	52 (42)			
Methadone started	5 (4)			
Bup/Nx discontinued	8 (7)			
Fetal exposure to illicit drugs	93 (76)			
Benzodiazepines	71 (58)			
Opioids	58 (47)			
Cannabis	39 (32)			
Stimulants	32 (26)			
Other drugs	6 (5)			
Documented illicit drug use in the III trimester	52 (48)			
Fetal exposure to alcohol	24 (21)			
Fetal exposure to smoking	117 (95)	3379 (8)	248.52 (105.81 to 546.73)	<0.001
Fetal exposure to psychopharmaca	72 (59)			
SSRI or SNRI	29 (24)			
Other antidepressants	19 (15)			
Antipsychotics	37 (30)			
Benzodiazepines	53 (43)			

a: Reference perinatal data are taken from the Perinatal Statistics 2022, compiled by the Finnish Institute for Health and Welfare.

b: Reference data are taken from the Register of Congenital Malformations 2021, compiled by the Finnish Institute for Health and Welfare.

c: One child with a combination of pyloric stenosis, anal stenosis, perineal groove and bilateral club finger.

*pH* potential of hydrogen, *g* grams, *cm* centimeters, *HC* head circumference.

*SD* standard deviation. The growth parameters expressed as SDs are specific to parity, single or twin pregnancies, gestational age, and gender, compared to general Finnish newborns.

*SGA* small for gestational age, *NOWS* neonatal opioid withdrawal syndrome, *OMT* opioid maintenance treatment, *Bup/Nx* Buprenorphine ± naloxone, *SSRI or SNRI* Selective serotonin or serotonin–norepinephrine reuptake inhibitors.

Occasional cases of congenital diagnoses were observed (Table 1). None of these children had reported alcohol exposure (*p* = *0.342*), nor statistically significant links with maternal OMT, psychiatric or epilepsy pharmacotherapy, illicit drug use, or smoking (data not shown, DNS). None of the newborns had chromosomal abnormalities or thyroidal dysfunction. One child was later diagnosed with Marfan syndrome (Q87.4) and one with long QT syndrome (I49.8).

Of the 123 newborns, 58 (47%) were exposed to maternal OMT throughout their gestation and were included in our previous studies,^20–22^ where more detailed data on their mothers are reported. The remaining 65 mothers showed no significant differences from the previously reported group, except for lacking reliable data on the extent of substance use during pregnancy for 59 mothers before starting OMT and eight mothers after discontinuing it. Of the children, 76% were exposed to maternal illicit substance use concomitantly with OMT (Table 1). Of all 123 children, 76 (62%) had known polysubstance exposure, most often to 2-4 substances. Among those whose mothers initiated OMT during pregnancy, 52/57 (91%) were prenatally exposed to multiple substances. Only 6/99 (6%) of mothers who gave drug screens through pregnancy had consistently negative results. In 24 (20%) of the cases, the frequency of screening was inadequate to assess fetal drug exposure properly. All neonates were monitored for NOWS using Finnegan scoring, with 69 (56%) requiring pharmacological treatment for NOWS with morphine only or with phenobarbital.

### At the age of four

**Fig. 2:**
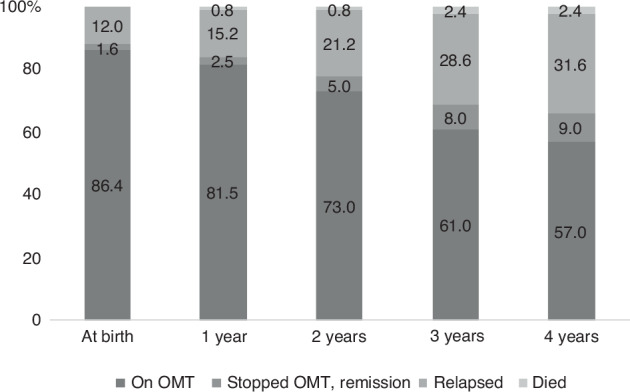
Maternal adherence to opioid maintenance treatment after delivery during the 4-year follow-up. At birth, 88% of mothers were in opioid maintenance treatment or remission. Over 4 years, those in treatment decreased, while the number in remission increased, and one in three relapsed.

Parental opioid use disorder (OUD) influenced the children’s living arrangements (Table 2). After delivery, 108 (88%) mothers continued OMT or were in remission without maintenance medication (Fig. 2). During the follow-up, 42 (34%) relapsed, and three of them died. Six fathers assumed custody of the child, yet 37 (30%) of the children were in foster care or adopted by the age of four.

**Table 2:** Characteristics related to children at the age of four.

*n* (%)	Cohort *n* = 123	Finland^a^ *n* = 50,457	OR (95% CI)
Father known	114 (93)		
Substance use problem	102 (90)		
On OMT	46 (45)		
Lived with family	36 (32)		
Died before child’s fifth birthday	5 (4)		
Domestic violence	24 (20)		
Living			
With biological parent/s	86 (70)		
In foster care or adoption	37 (30)		
Never lived with biological parents	7 (6)		
Diagnosed chronic somatic diseases	47 (38)		
Strabismus	13 (10.6)	904 (1.8)	6.48 (3.63 to 11.55)
Growth hormone deficiency	3 (2.4)	31 (0.06)	40.67 (12.27 to 134.82)
Hepatitis C	4 (3.3)	4 ( < 0.01)	423.97 (104.81 to 1715.10)
Asthma	7 (5.7)	1237 (2.5)	2.40 (1.12 to 5.16)
Atopic dermatitis	17 (13.8)	875 (1.7)	9.09 (5.42 to 15.23)
Obstipation	8 (6.5)	135 (0.3)	25.93 (12.42 to 54.15)
Obesity	10 (8.1)	58 (0.1)	76.90 (38.34 to 154.24)

a: The ICD-10 diagnosis reference data are taken from the Hospital Discharge Register maintained by the Finnish Institute for Health and Welfare. These data include hospital care for Finnish children aged four in 2022 (*n* = 50,457).

*OMT* opioid maintenance treatment.

Among the 123 children, 38% had at least one chronic somatic disease unrelated to congenital diagnoses (Table 2). Of these, 26 (55%) had one, 16 (34%) had two, and 2 (5%) had three diagnoses. The most common combination was atopic dermatitis together with constipation, affecting five children. Of the three with short stature, two had isolated growth hormone deficiency and one constitutionally short stature. All three children received growth hormone therapy. Hepatitis C was diagnosed in four children. Among the mothers, 58 (47%) had detectable hepatitis C viruses (HCV) in their bloodstream during pregnancy, and one mother was diagnosed shortly after delivery, resulting in a hematogenous transmission rate of 6.8%. Of the four affected children, three were delivered via cesarean section and one was born vaginally.

The *Lene* screening test was available for 116 (94%) of the cohort (Table 3). Developmental difficulties were significantly more prevalent in the study group than in the controls (*OR 2.15, 95% CI 1.41 to 3.28*), often affecting multiple domains (*OR 2.04, 95% CI 1.24 to 3.36*). Males faced more often overall difficulties, particularly with language skills (*OR 5.45, 95% CI 2.93 to 10.11*), while females had more motor-perceptual issues (*OR 2.22, 95% CI 1.01 to 4.86*). Males unable to form two-word sentences by age two had significantly more often linguistic difficulties on the *Lene* test (*p* = *0.002*). Subgroup analysis revealed that fetal exposure to illicit opioids correlated with a lower chance of passing the *Lene* test, particularly in the language domain among females (*p* = *0.032*). All eight children with reported fetal alcohol exposure had challenges on *Lene*, but only one had fetal alcohol spectrum disorder diagnosis (FASD, Q86.0).

**Table 3:** Developmental difficulties in *Lene* test^a^ at age four.

	Cohort, *n* (%)			Reference, *n* (%)			Cohort vs. reference, OR (95% CI)		
	All (*n* = 116)	Males (*n* = 61)	Females (*n* = 55)	All (*n* = 434)	Males (*n* = 197)	Females (*n* = 237)	All	Males	Females
Any difficulties in *Lene*	54 (47)	39 (64)	15 (27)	125 (29)	80 (41)	45 (19)	2.15 (1.4 to 3.3)	2.59 (1.4 to 4.7)	1.60 (0,8 to 3.2)
Difficulties in language domain	42 (36)	34 (56)	8 (15)	53 (12)	37 (19)	16 (7)	4.08 (2.54 to 6.56)	5.45 (2.93 to 10.1)	2.35 (0.95 to 5.81)
Difficulties in motor-perceptual domain	30 (26)	19 (31)	11 (20)	73 (17)	49 (25)	24 (10)	1.73 (1.06 to 2.80)	1.37 (0.73 to 2.57)	2.22 (1.01 to 4.86)
Difficulties in attention-behavioral domain	28 (24)	19 (31)	9 (16)	84 (19)	60 (31)	24 (10)	1.33 (0.81 to 2.16)	1.03 (0.56 to 1.92)	1.74 (0.76 to 3.98)
Difficulties in 1 domain	33 (28)			64 (15)			2.30 (1.42 to 3.73)		
Difficulties in ≥2 domains	29 (25)			61 (14)			2.04 (1.24 to 3.36)		

a: *Lene* test is a neurodevelopmental screening method, which includes age-appropriate assessments, derived from various standardized tests. Reference group data for *Lene* test were obtained from randomly selected health clinic data of Finnish children representing the general population.^23^

As shown in Table 4, compared to the general Finnish population of four-year-olds, the prevalence of speech and language developmental disorder diagnosis (F80) was significantly higher in the cohort (*OR 8.97, 95% CI 5.64 to 14.28*), especially among males (*p* < *0.001*), and those with fetal exposure to methadone OMT (*p* = *0.004*), or the combination of buprenorphine OMT and additional opioids (*p* = *0.011*). Attention-deficit hyperactivity disorder (ADHD, F90) was notably common in the cohort. Although five mothers had ADHD diagnoses, none of their children did. Instead, ADHD in children was linked to domestic violence (*p* = *0.023*), with risk further elevated when domestic violence was combined with father’s substance use (*OR 3.86, 95% CI 1.14 to 13.03, p* = *0.033*). Conduct disorders (F91), mixed disorders of conduct and emotions (F92), emotional disorders with onset specific to childhood (F93), and disorders of social functioning with onset specific to childhood and adolescence (F94), as well as FASD diagnoses, were also significantly more common in the study cohort than in the general population.

**Table 4:** Developmental and behavioral ICD-10 diagnoses at age four

	Cohort (*n* = 123) ^a^			Reference (*n* = 50,457)^b^			Cohort vs. reference, OR (95% CI)		
Diagnosed, *n* (%)	All	M	F	All	M	F	All	M	F
F80 Specific developmental disorders of speech and language	22 (17.9)	19 (29.3)	3 (5.2)	1196 (2.4)	864 (3.3)	332 (1.4)	8.97 (5.64 to 14.28)	11.97 (6.98 to 20.57)	3.98 (1.24 to 12.79)
F82 Specific developmental disorder of motor function	1 (0.8)	1 (1.5)	0	108 (0.2)	80 (0.3)	28 (0.1)	3.82 (0.53 to 27.59)	5.04 (0.69 to 36.79)	N/A
F83 Mixed specific developmental disorders	3 (2.4)	3 (4.6)	0	589 (1.2)	410 (1.6)	179 (0.7)	2.12 (0.67 to 6.68)	3.01 (0.94 to 9.62)	N/A
F84 Pervasive developmental disorders	1 (0.8)	1 (1.5)	0	343 (0.7)	254 (1.0)	89 (0.4)	1.20 (0.17 to 8.60)	1.58 (0.22 to 11.41)	N/A
Q86.0 Fetal alcohol syndrome	1 (0.8)	0	1 (1.7)	6 (0.01)	5 (0.02)	1 (0.004)	68.92 (8.24 to 576.78)	N/A	430.91 (26.63 to 6973.84)
F90 Attention-deficit hyperactivity disorders	14 (11.4)	7 (10.8)	7 (12.1)	124 (0.2)	92 (0.4)	32 (0.1)	52.14 (29.08 to 93.46)	33.85 (15.05 to 76.13)	105.22 (44.40 to 249.33)
F91 Conduct disorders	3 (2.4)	0	3 (5.2)	15 (0.03)	11 (0.04)	4 (0.02)	84.07 (24.03 to 294.16)	N/A	334.90 (73.23 to 1531.49)
F92 Mixed disorders of conduct and emotions	3 (2.4)	3 (4.6)	0	6 (0.01)	4 (0.02)	2 (0.01)	210.21 (51.97 to 850.29)	313.19 (68.66 to 1428.49)	N/A
F93 Emotional disorders with onset specific to childhood	3 (2.4)	2 (3.1)	1 (1.7)	62 (0.1)	30 (0.1)	32 (0.1)	20.32 (6.29 to 65.63)	27.37 (6.40 to 116.98)	13.45 (1.81 to 100.10)
F94 Disorders of social functioning with onset specific to childhood	7 (5.7)	4 (6.1)	3 (5.2)	38 (0.08)	16 (0.06)	22 (0.09)	80.07 (35.04 to 182.97)	106.06 (34.46 to 326.39)	60.85 (17.70 to 209.20)

a: The study cohort of 123 children included 65 males (M) and 58 females (F)

b: The ICD-10 diagnosis reference data is sourced from the Hospital Discharge Register maintained by the Finnish Institute for Health and Welfare.

This data includes Finnish children aged four in 2022, *n* = 50,457, males (M) 25,894 and females (F) 24,563.

Subgroup analyses, aside from those previously mentioned, revealed no significant associations between developmental, behavioral, or emotional ICD-10 diagnoses and several factors. These factors included: (1) fetal exposure to maternal maintenance medication, drugs, alcohol, or smoking; (2) maternal psychiatric diagnoses; (3) prematurity; (4) birth weight of less than 2500 g; (5) being small for gestational age (SGA); (6) head circumference smaller than -2 SD at birth, at age two, or age four; (7) growth measurements (height, weight, and head circumference) at age four; (8) somatic diseases; and (9) living with biological parent/s or in out-of-home care.

## Discussion

This study shows that prenatal exposure to maternal OUD and OMT is linked to developmental, behavioral, and emotional challenges in children at the age of four. These findings are, on one hand, in line with clinical experience and some previous studies,^25–27^ but on the other hand, in contrast with some others suggesting no neurodevelopmental harm from intrauterine opioid exposure.^15–18^ In these latter studies, the follow-up may have been too short to detect developmental problems, which may not emerge until later in childhood, despite originating during the fetal period.

Prenatal exposure to opioids may directly affect neural development, and thus lead to challenges in later development.^28,29^ However, beyond the direct effects of OMT, other factors may contribute, such as genetic predisposition,^30^ or exposures to additional harmful substances. In this study, all cohort children were exposed to opioids throughout the gestation. Those whose mothers initiated OMT before conception were likely exposed in a more controlled manner than those whose mothers began treatment during pregnancy, though outcomes were not significantly different. Alarmingly, 76% of the cohort´s mothers used illicit drugs during pregnancy, complicating the isolation of individual substance effects. Nonetheless, combined fetal exposure to multiple substances was significantly associated with later developmental difficulties. Maternal methadone treatment and additional opioid use during buprenorphine OMT were particularly linked to speech and language disorders.

Despite the high prevalence of reported illicit drug use during pregnancy, the actual use may have been even higher. Child urine and meconium samples indicate exposures only to a limited extent, and the data from mothers were based on the patient self-reports and voluntary urine tests, when the fear of child protection services involvement may have hindered truthful reporting. The same applied to alcohol, especially as detection methods for fetal alcohol exposure were less advanced when the cohort was born. Therefore, although FASD was more common in the cohort than in the general population, it may have been underdiagnosed. Additionally, children may have been too young for diagnosis, as the diagnosis of FASD is often assessed at school age when learning and executive functioning difficulties emerge. Eight children with known fetal alcohol exposure displayed challenges on the *Lene* test at age four, though only one was diagnosed with FASD. Thus, further follow-up will reveal if additional FASD diagnoses emerge.

The potential impact of smoking exposure could not be fully assessed due to data limitations in the reference group. Within the cohort group, maternal smoking status, and its connection to the child’s screening test results and ICD-10 diagnoses were available, but in the reference group, only the overall smoking prevalence among mothers was known, without any link to the child’s *Lene* test or ICD-10 diagnoses.

In addition to fetal exposures, the postnatal environment may naturally have exacerbating or buffering effects on neurodevelopment and behavior. Parent-child interaction, parental stress and mental challenges, social environment, and financial difficulties may all play roles. Early ACEs, such as low parental sensitivity to a child’s needs and mood, limited responsiveness, and reduced positive interaction, may impair development.^26,31^ Furthermore, NOWS-related newborn irritability can create a negative cycle, where parental frustration may lead to more negative than positive responses toward the child.^31^ Importantly, however, OUD does not necessarily imply poor parenting skills. Many parents benefit from strong support, which can help them channel their potential and resources into effective childcare and parenting, with nurturing care acting as a buffer against negative outcomes.^32^

Although maternal psychiatric comorbidities may elevate risks for adverse outcomes in children by influencing parenting style^33^ or via genetics, psychiatric comorbidity in this cohort was not statistically significantly linked to developmental or behavioral diagnoses of the children. For instance, none of the children whose mothers had ADHD received an ADHD diagnosis. Instead, ADHD in children was statistically significantly associated with domestic violence and fathers’ substance use disorder, underscoring the impact of an unsafe environment. Additionally, maternal psychiatric diagnoses may have been underreported in this cohort, as these conditions may have been assessed in primary care and not recorded at the tertiary hospital. ADHD may also be underdiagnosed in women in general. Notably, ADHD diagnoses in children were common in this cohort, even though diagnosing ADHD at four years of age is atypical. This early diagnosis may indicate that either the children with ADHD had a more severe form than in general, or that more intensive follow-up enabled earlier identification of the diagnosis.

In this study, neurodevelopmental outcomes did not significantly differ between children living with biological parent/s or placed early in foster care or adoption, possibly reflecting the heterogeneity within these groups. Children placed early into foster care or being adopted may have experienced greater fetal substance exposures, as their mothers tend to come from more complicated backgrounds. As a balancing issue, placement in stable homes offers basic security early on. However, some children face multiple foster placements, leading to recurrent losses of caring adults, which can be traumatic. In turn, children initially living with parents may have milder fetal exposures, and some parents find motivation and strength to stabilize their lives by having a child. However, parents’ relapses lead their children to suffer from unstable conditions. Thus, this study does not support a definitive preference for parental care, foster care, or adoption. It does, however, emphasize the importance of providing a safe and stable environment with consistent, caring adults.

Several additional factors may naturally be involved in development and behavior difficulties. For example, maturation rates differ between sexes, with male sex on average developing more slowly than female sex.^23,34^ Male children have also been suggested to be more vulnerable to fetal exposure to maternal OMT than female children.^11,35^ Consistent with these prior studies, males in this cohort faced more adverse outcomes. Interestingly, among females, maternal concomitant substance use during pregnancy was linked to greater developmental challenges. In turn, prematurity, SGA and pharmacotherapy for NOWS showed no significant associations with adverse outcomes.

In addition to developmental and emotional challenges, children in the study group exhibited a higher prevalence of chronic somatic illnesses compared to their Finnish same-aged peers. Diagnoses typically assessed in tertiary care settings in Finland -such as strabismus, growth hormone deficiency, asthma, and hepatitis C infection- probably reflect true differences. While strabismus and hepatitis C are documented complications associated with these types of patient groups, we found no literature linking growth hormone deficiency to fetal opioid exposure. This phenomenon is intriguing but may be coincidental within this cohort and warrants further investigation.

Conditions like atopic dermatitis, obstipation, and obesity are more commonly diagnosed in primary care settings. Consequently, these diagnoses may be underrepresented in the Finnish national hospital register compared to the study cohort. However, obesity may genuinely be more prevalent among children of parents with OUD, as demonstrated in our recent work on OMT-exposed toddlers at the age of two.^21^ The excessive weight gain observed in toddlers may have multifactorial causes, highlighting the need for parental guidance on healthy nutrition and additional parenting support when aiming at preventing long-term health complications.

Our cohort was relatively large and comprehensive, given the usual challenges in substance research, such as social stigma, recruitment problems, dropouts and adherence difficulties. Pregnancies were closely monitored, deliveries planned, and childcare and follow-up provided in standardized conditions, free of charge, along with social support aimed at promoting treatment adherence. The *Lene* test results were objective, and ICD-10 diagnoses were assessed by specialists.

Despite these strengths, the cohort size remained limited, potentially impacting the study’s power to detect all clinically significant factors and complex relationships between the variables. Moreover, although the dropout rates were relatively low, and the reason in many cases moving to another location rather than social or health issues, missing data from these cases may have influenced the results. Additionally, there are limitations with the reference groups, such as the lack of data on maternal alcohol or illicit substance use during pregnancy. Furthermore, although the reference and study groups were the same age at the time of their *Lene* screening tests and thus comparable, they were tested at different times, which may have influenced the results. The study also did not assess fathers’ psychiatric diagnoses, parental developmental difficulties, parental education, socioeconomic status, or ACEs, which is an obvious limitation that warrants investigation in future research. Finally, identifying the specific roles of variables associated with poorer outcomes remains challenging, as these factors are often interrelated, such as exposure to illicit drugs and unfavorable environment.

## Conclusion

Children with in-utero exposure to maternal OMT are at increased risk for developmental, behavioral, emotional, and health challenges compared to general population. These children often face multiple risk factors that may influence development, with compounded risks leading to greater likelihood of impairments later in life. Every child deserves the opportunity for healthy development, including behavioral and language skills, mental well-being, stable environment and supportive caregivers. Early identification and rehabilitation are essential for fostering better long-term outcomes for this vulnerable group.

## Data Availability

Due to Finnish data protection laws, the authors cannot provide access to the data on children with in-utero exposure to maternal OMT. Access to Finnish register data can be requested from the Finnish Social and Health Data Permit Authority (Findata; www.findata.fi).

## References

[1] *Guidelines for the Identification and Management of Substance Use and Substance Use Disorders in Pregnancy*. World Health Organization (2014). 24783312

[2] Hunt, R. W., Tzioumi, D., Collins, E. & Jeffery, H. E. Adverse neurodevelopmental outcome of infants exposed to opiate in-utero. *Early Hum. Dev.* **84**, 29–35 (2008). 17728081 10.1016/j.earlhumdev.2007.01.013

[3] Kim, H. M. et al. Preschool language development of children born to women with an opioid use disorder. *Child Basel Switz.* **8**, 268 (2021). 10.3390/children8040268 PMC8066299 33807265

[4] Lee, S. J., Bora, S., Austin, N. C., Westerman, A. & Henderson, J. M. T. Neurodevelopmental outcomes of children born to opioid-dependent mothers: a systematic review and meta-analysis. *Acad. Pediatr.* **20**, 308–318 (2020). 31734383 10.1016/j.acap.2019.11.005

[5] Yeoh, S. L., Eastwood, J. & Wright, I. M. Cognitive and motor outcomes of children with prenatal opioid exposure: a systematic review and meta-analysis. *JAMA Netw. Open* **2**, e197025 (2019). 31298718 10.1001/jamanetworkopen.2019.7025 PMC6628595

[6] Aslaksen, A. K. et al. Children had increased risks of impaired motor and visual-motor skills after prenatal exposure to opioid maintenance therapy. *Acta Paediatr. Oslo Nor. 1992* **113**, 1331–1339 (2024). 10.1111/apa.17175 38415880

[7] Fill, M. M. A. et al. Educational disabilities among children born with neonatal abstinence syndrome. *Pediatrics* **142**, e20180562 (2018). 30166364 10.1542/peds.2018-0562 PMC6947655

[8] Miller, J. S., Anderson, J. G., Erwin, P. C., Davis, S. K. & Lindley, L. C. The effects of neonatal abstinence syndrome on language delay from birth to 10 years. *J. Pediatr. Nurs.* **51**, 67–74 (2020). 31923742 10.1016/j.pedn.2019.12.011

[9] Hall, E. S., McAllister, J. M. & Wexelblatt, S. L. Developmental disorders and medical complications among infants with subclinical intrauterine opioid exposures. *Popul Health Manag* **22**, 19–24 (2019). 29893624 10.1089/pop.2018.0016 PMC6386081

[10] Oei, J. L. et al. Neonatal abstinence syndrome and high school performance. *Pediatrics* **139**, e20162651 (2017). 28093465 10.1542/peds.2016-2651

[11] Nygaard, E., Moe, V., Slinning, K. & Walhovd, K. B. Longitudinal cognitive development of children born to mothers with opioid and polysubstance use. *Pediatr. Res.* **78**, 330–335 (2015). 25978800 10.1038/pr.2015.95 PMC4539602

[12] Welton, S., Blakelock, B., Madden, S. & Kelly, L. Effects of opioid use in pregnancy on pediatric development and behaviour in children older than age 2: Systematic review. *Can. Fam. Physician Med Fam. Can.* **65**, e544–e551 (2019). PMC6907383 31831504

[13] Leverett, S. D. et al. Associations between parenting and cognitive and language abilities at 2 years of age depend on prenatal exposure to disadvantage. *J. Pediatr.* **276**, e114289 (2025). 10.1016/j.jpeds.2024.114289 PMC11927132 39233119

[14] Ornoy, A., Segal, J., Bar-Hamburger, R. & Greenbaum, C. Developmental outcome of school-age children born to mothers with heroin dependency: importance of environmental factors. *Dev. Med Child Neurol.* **43**, 668–675 (2001). 11665823 10.1017/s0012162201001219

[15] Sundelin Wahlsten, V. & Sarman, I. Neurobehavioural development of preschool-age children born to addicted mothers given opiate maintenance treatment with buprenorphine during pregnancy. *Acta Paediatr. Oslo Nor. 1992* **102**, 544–549 (2013). 10.1111/apa.12210 23432078

[16] Kang, J. et al. Prenatal opioid exposure and subsequent risk of neuropsychiatric disorders in children: nationwide birth cohort study in South Korea. *BMJ* **385**, e077664 (2024). 38658035 10.1136/bmj-2023-077664 PMC11040462

[17] Kaltenbach, K. et al. Prenatal exposure to methadone or buprenorphine: Early childhood developmental outcomes. *Drug Alcohol Depend.* **185**, 40–49 (2018). 29413437 10.1016/j.drugalcdep.2017.11.030 PMC5906792

[18] Bierce, L., Tabachnick, A. R., Eiden, R. D., Dozier, M. & Labella, M. H. A 12-month follow-up of infant neurodevelopmental outcomes of prenatal opioid exposure and polysubstance use. *Neurotoxicol. Teratol.* **97**, e107176 (2023). 10.1016/j.ntt.2023.107176 PMC10198960 37054901

[19] Levine, T. A., Davie-Gray, A., Kim, H. M., Lee, S. J. & Woodward, L. J. Prenatal methadone exposure and child developmental outcomes in 2-year-old children. *Dev. Med. Child Neurol.* **63**, 1114–1122 (2021). 33462809 10.1111/dmcn.14808

[20] Kanervo, M. M., Tupola, S. J., Nikkola, E. M., Rantakari, K. M. & Kahila, H. K. Buprenorphine-naloxone, buprenorphine, and methadone throughout pregnancy in maternal opioid use disorder. *Acta Obstet. Gynecol. Scand.* **102**, 313–322 (2023). 36562462 10.1111/aogs.14497 PMC9951318

[21] Kanervo, M. et al. Intrauterine exposure to maternal opioid maintenance treatment and associated risk factors may impair child growth. *Acta Paediatr. Oslo Nor. 1992* **113**, 1579–1591 (2024). 10.1111/apa.17198 38456564

[22] Kanervo, M., Tupola, S., Nikkola, E., Rantakari, K. & Kahila, H. Extended-release versus oral buprenorphine as opioid maintenance treatment during pregnancy-maternal and neonatal outcomes. *Eur. J. Obstet. Gynecol. Reprod. Biol.* **297**, 106–110 (2024). 38608352 10.1016/j.ejogrb.2024.04.003

[23] Valtonen, R., Ahonen, T., Lyytinen, P. & Lyytinen, H. Co-ocurrence of developmental delays in a screening study of 4-year-old Finnish children. *Dev. Med. Child Neurol.* **46**, 436–443 (2004). 15230455 10.1017/s0012162204000726

[24] Finnegan, L. P., Connaughton, J. F. J., Kron, R. E. & Emich, J. P. Neonatal abstinence syndrome: assessment and management. *Addict. Dis.* **2**, 141–158 (1975). 1163358

[25] Jaekel, J. et al. Emotional and behavioral trajectories of 2 to 9 years old children born to opioid-dependent mothers. *Res. Child Adolesc. Psychopathol.* **49**, 443–457 (2021). 33433780 10.1007/s10802-020-00766-w PMC7943531

[26] Koponen, A. M. et al. Prenatal substance exposure, adverse childhood experiences and diagnosed mental and behavioral disorders - A longitudinal register-based matched cohort study in Finland. *SSM - Popul Health* **11**, e100625 (2020). 10.1016/j.ssmph.2020.100625 PMC7358713 32685656

[27] Irner, T. B. Substance exposure in utero and developmental consequences in adolescence: A systematic review. *Child Neuropsychol.* **18**, 521–549 (2012). 22114955 10.1080/09297049.2011.628309

[28] Monnelly, V. J. et al. Prenatal methadone exposure is associated with altered neonatal brain development. *NeuroImage Clin.* **18**, 9–14 (2018). 29326869 10.1016/j.nicl.2017.12.033 PMC5760461

[29] Merhar, S. L. et al. Prenatal opioid exposure is associated with smaller brain volumes in multiple regions. *Pediatr. Res.* **90**, 397–402 (2021). 33177677 10.1038/s41390-020-01265-w PMC8110593

[30] Wachman, E. M. et al. Association of maternal and infant variants in PNOC and COMT genes with neonatal abstinence syndrome severity. *Am. J. Addict.* **26**, 42–49 (2017). 27983768 10.1111/ajad.12483 PMC5206487

[31] Konijnenberg, C., Sarfi, M. & Melinder, A. Mother-child interaction and cognitive development in children prenatally exposed to methadone or buprenorphine. *Early Hum. Dev.* **101**, 91–97 (2016). 27614330 10.1016/j.earlhumdev.2016.08.013

[32] Beldick, S. R., Rohde, J. F., Short, V. L., Abatemarco, D. & Goyal, N. K. Pediatric primary care diagnosis patterns among children with intrauterine opioid exposure. *J. Health Care Poor Underserved* **34**, 161–179 (2023). 37464487 10.1353/hpu.2023.0011 PMC10483573

[33] Winstanley, E. L. & Stover, A. N. The impact of the opioid epidemic on children and adolescents. *Clin. Ther.* **41**, 1655–1662 (2019). 31303278 10.1016/j.clinthera.2019.06.003 PMC7017799

[34] Etchell, A. et al. A systematic literature review of sex differences in childhood language and brain development. *Neuropsychologia* **114**, 19–31 (2018). 29654881 10.1016/j.neuropsychologia.2018.04.011 PMC5988993

[35] Skumlien, M., Ibsen, I. O., Kesmodel, U. S. & Nygaard, E. Sex differences in early cognitive development after prenatal exposure to opioids. *J. Pediatr. Psychol.* **45**, 475–485 (2020). 32324876 10.1093/jpepsy/jsaa008 PMC7233842

